# (4-Meth­oxy­phen­yl)(4-methyl­cyclo­hex­yl)methanone

**DOI:** 10.1107/S1600536812037373

**Published:** 2012-09-05

**Authors:** Wei Liu, Lida Tang

**Affiliations:** a Graduate School, Tianjin Medical University, Tianjin 300070, People’s Republic of China; b Tianjin Key Laboratory of Molecular Design & Drug Discovery, Tianjin Institute of Pharmaceutical Research, Tianjin 300193, People’s Republic of China; c State Key Laboratory of Drug Delivery Technology and Pharmacokinetics, Tianjin Institute of Pharmaceutical Research, Tianjin 300193, People’s Republic of China

## Abstract

The title compound, C_15_H_20_O_2_, crystallizes with two independent mol­ecules of similar geometry in the asymmetric unit. The cyclo­hexyl ring adopts a chair conformation in each mol­ecule. In the crystal, mol­ecules related by translation are linked into chains along the *a* axis *via* weak C—H⋯O inter­actions.

## Related literature

For the anti­hyperglycemic activity of SGLT2 inhibitors, see: Shao *et al.* (2011 ▶); Zhao *et al.* (2011 ▶). For related structures, see: Meng *et al.* (2012 ▶); Wang *et al.* (2011 ▶).
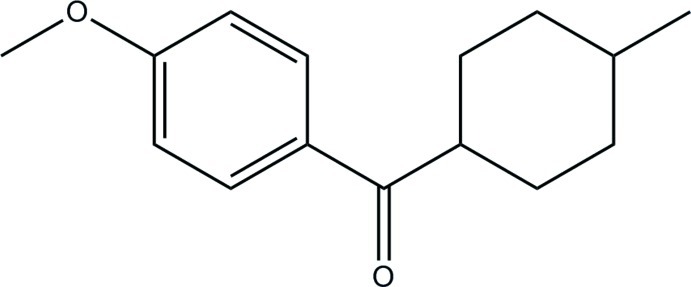


## Experimental

### 

#### Crystal data

C_15_H_20_O_2_
*M* *_r_* = 232.31
Triclinic, 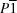

*a* = 5.7003 (18) Å
*b* = 7.279 (2) Å
*c* = 30.76 (1) Å
α = 96.700 (9)°
β = 94.380 (16)°
γ = 93.332 (12)°
*V* = 1261.0 (7) Å^3^
*Z* = 4
Mo *K*α radiation
μ = 0.08 mm^−1^
*T* = 113 K
0.20 × 0.18 × 0.12 mm

#### Data collection

Rigaku Saturn CCD area-detector diffractometer
Absorption correction: multi-scan (*CrystalClear*; Rigaku/MSC, 2009 ▶) *T*_min_ = 0.984, *T*_max_ = 0.991
12908 measured reflections
5924 independent reflections
3739 reflections with *I* > 2σ(*I*)
*R*_int_ = 0.038

#### Refinement

*R*[*F* ^2^ > 2σ(*F* ^2^)] = 0.043
*wR*(*F* ^2^) = 0.120
*S* = 1.03
5924 reflections
311 parameters
H-atom parameters constrained
Δρ_max_ = 0.35 e Å^−3^
Δρ_min_ = −0.18 e Å^−3^

### 

Data collection: *CrystalClear* (Rigaku/MSC, 2009 ▶); cell refinement: *CrystalClear*; data reduction: *CrystalClear*; program(s) used to solve structure: *SHELXS97* (Sheldrick, 2008 ▶); program(s) used to refine structure: *SHELXL97* (Sheldrick, 2008 ▶); molecular graphics: *SHELXTL* (Sheldrick, 2008 ▶); software used to prepare material for publication: *SHELXTL*.

## Supplementary Material

Crystal structure: contains datablock(s) I, global. DOI: 10.1107/S1600536812037373/cv5337sup1.cif

Structure factors: contains datablock(s) I. DOI: 10.1107/S1600536812037373/cv5337Isup2.hkl

Supplementary material file. DOI: 10.1107/S1600536812037373/cv5337Isup3.cml

Additional supplementary materials:  crystallographic information; 3D view; checkCIF report

## Figures and Tables

**Table 1:** Hydrogen-bond geometry (Å, °)

*D*—H⋯*A*	*D*—H	H⋯*A*	*D*⋯*A*	*D*—H⋯*A*
C9—H9⋯O2^i^	1.00	2.57	3.4694 (18)	150
C24—H24⋯O4^ii^	1.00	2.60	3.5244 (18)	154

Symmetry codes: (i) 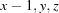; (ii) 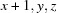.

**Fig. 1.**
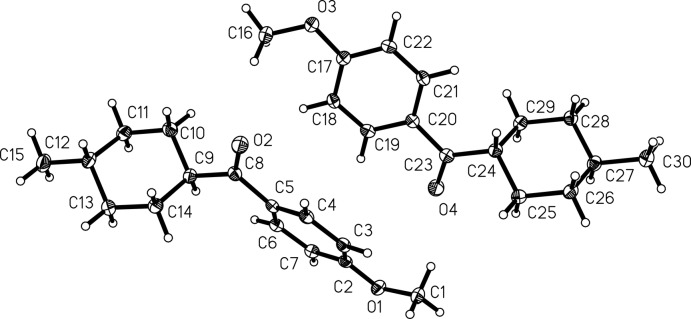
Two independent molecules of the title compound, with displacement ellipsoids drawn at the 40% probability level.

## supplementary crystallographic information

### Comment

SGLT2 inhibitors represent an emerging class of promising antihyperglycemic agents, and a lot of drug candidates are now filed or in phase III clinical trials. During the study of saturated ring-bearing SGLT2 inhibitors (Shao *et al.*, 2011; Zhao *et al.*, 2011) the title compound was prepared, which is a key intermediate for the synthetic procedure.

In title compound, C_15_H_20_O_2_, crystallizes with two independent molecules of similar geometry in the asymmetric unit. All bond lengths and angles are normal and in a good agreement with those reported previously for related compounds (Meng *et al.*, 2012; Wang *et al.*, 2011). The cyclohexyl rings (C9—C14 and C24—C29) adopt chair confirmation. In the crystal, the molecules related by translation along the *a* axis are linked into chains *via* weak C—H···O interactions.

### Experimental

A mixture of 2.46 g (10 mmol) of ethyl 3-(4-methoxyphenyl)-1*H*-pyrazole-5-carboxylate, 3.76 g (20 mmol) of 1,2-dibromoethane, 5.53 g (40 mmol) of K_2_CO_3_ in 30 ml of dried acetonitrile was refluxed overnight. On cooling, the reaction mixture was filtered and poured into 200 ml of brine. The resulting mixture was extracted with three 50-ml of dichloromethane, and combined extracts were washed with saturated brine, dried over Na_2_SO_4_ and evaporated on a rotary evaporator to afford the crude product as brown solid, which was purified by column chromatography to yield the pure product as colorless crystals (yield 88%). The single crystals suitable for single-crystal X-ray diffraction was obtained by slow evaporation at room temperature of a solution of the title compound in dichloromethane/hexane (1/5) (m.p.88–90 Celsius degree).

### Refinement

All H atoms bonded on carbon were found on difference maps, with C–H = 0.95–1.00, and included in the final cycles of refinement using a riding model, with *U*_iso_(H) = 1.2*U*_eq_(C) and 1.5*U*_eq_(C) for the methyl H atoms.

### Crystal data

**Table:**

C_15_H_20_O_2_	*Z* = 4
*M**_r_* = 232.31	*F*(000) = 504
Triclinic, *P*1	*D*_x_ = 1.224 Mg m^−^^3^
Hall symbol: -P 1	Mo *K*α radiation, λ = 0.71073 Å
*a* = 5.7003 (18) Å	Cell parameters from 4144 reflections
*b* = 7.279 (2) Å	θ = 2.0–27.9°
*c* = 30.76 (1) Å	µ = 0.08 mm^−^^1^
α = 96.700 (9)°	*T* = 113 K
β = 94.380 (16)°	Prism, colourless
γ = 93.332 (12)°	0.20 × 0.18 × 0.12 mm
*V* = 1261.0 (7) Å^3^	

### Data collection

**Table:**

Rigaku Saturn CCD area-detector diffractometer	5924 independent reflections
Radiation source: rotating anode	3739 reflections with *I* > 2σ(*I*)
Multilayer monochromator	*R*_int_ = 0.038
Detector resolution: 14.63 pixels mm^-1^	θ_max_ = 27.9°, θ_min_ = 2.0°
ω and φ scans	*h* = −7→7
Absorption correction: multi-scan (*CrystalClear*; Rigaku/MSC, 2009)	*k* = −9→9
*T*_min_ = 0.984, *T*_max_ = 0.991	*l* = −39→40
12908 measured reflections	

### Refinement

**Table:**

Refinement on *F*^2^	Primary atom site location: structure-invariant direct methods
Least-squares matrix: full	Secondary atom site location: difference Fourier map
*R*[*F*^2^ > 2σ(*F*^2^)] = 0.043	Hydrogen site location: inferred from neighbouring sites
*wR*(*F*^2^) = 0.120	H-atom parameters constrained
*S* = 1.03	*w* = 1/[σ^2^(*F*_o_^2^) + (0.0506*P*)^2^] where *P* = (*F*_o_^2^ + 2*F*_c_^2^)/3
5924 reflections	(Δ/σ)_max_ = 0.004
311 parameters	Δρ_max_ = 0.35 e Å^−^^3^
0 restraints	Δρ_min_ = −0.18 e Å^−^^3^

### Special details

**Table:**

Geometry. All e.s.d.'s (except the e.s.d. in the dihedral angle between two l.s. planes) are estimated using the full covariance matrix. The cell e.s.d.'s are taken into account individually in the estimation of e.s.d.'s in distances, angles and torsion angles; correlations between e.s.d.'s in cell parameters are only used when they are defined by crystal symmetry. An approximate (isotropic) treatment of cell e.s.d.'s is used for estimating e.s.d.'s involving l.s. planes.
Refinement. Refinement of *F*^2^ against ALL reflections. The weighted *R*-factor *wR* and goodness of fit *S* are based on *F*^2^, conventional *R*-factors *R* are based on *F*, with *F* set to zero for negative *F*^2^. The threshold expression of *F*^2^ > σ(*F*^2^) is used only for calculating *R*-factors(gt) *etc*. and is not relevant to the choice of reflections for refinement. *R*-factors based on *F*^2^ are statistically about twice as large as those based on *F*, and *R*- factors based on ALL data will be even larger.

### Fractional atomic coordinates and isotropic or equivalent isotropic displacement parameters (Å^2^)

**Table:**

	*x*	*y*	*z*	*U*_iso_*/*U*_eq_	
O1	0.19664 (16)	1.11167 (12)	0.33482 (3)	0.0263 (2)	
O2	0.46796 (15)	0.82156 (13)	0.14498 (3)	0.0305 (2)	
O3	0.77830 (16)	0.33602 (12)	0.17072 (3)	0.0275 (2)	
O4	0.52284 (16)	0.73059 (14)	0.35409 (3)	0.0334 (3)	
C1	0.4001 (2)	1.19400 (18)	0.36164 (4)	0.0295 (3)	
H1A	0.5278	1.1096	0.3598	0.044*	
H1B	0.3614	1.2175	0.3922	0.044*	
H1C	0.4506	1.3113	0.3513	0.044*	
C2	0.2251 (2)	1.05419 (16)	0.29179 (4)	0.0209 (3)	
C3	0.4298 (2)	1.08989 (16)	0.27130 (4)	0.0218 (3)	
H3	0.5619	1.1598	0.2870	0.026*	
C4	0.4368 (2)	1.02131 (16)	0.22748 (4)	0.0212 (3)	
H4	0.5772	1.0428	0.2135	0.025*	
C5	0.2447 (2)	0.92202 (16)	0.20324 (4)	0.0192 (3)	
C6	0.0383 (2)	0.89343 (16)	0.22415 (4)	0.0205 (3)	
H6	−0.0971	0.8298	0.2080	0.025*	
C7	0.0302 (2)	0.95706 (16)	0.26805 (4)	0.0213 (3)	
H7	−0.1094	0.9343	0.2821	0.026*	
C8	0.2704 (2)	0.84618 (17)	0.15690 (4)	0.0219 (3)	
C9	0.0566 (2)	0.80827 (17)	0.12401 (4)	0.0220 (3)	
H9	−0.0875	0.7917	0.1401	0.026*	
C10	0.0752 (2)	0.63558 (17)	0.09157 (4)	0.0272 (3)	
H10A	0.0743	0.5251	0.1075	0.033*	
H10B	0.2267	0.6453	0.0780	0.033*	
C11	−0.1284 (2)	0.61157 (18)	0.05559 (4)	0.0283 (3)	
H11A	−0.1074	0.5011	0.0346	0.034*	
H11B	−0.2787	0.5904	0.0690	0.034*	
C12	−0.1415 (2)	0.78128 (18)	0.03088 (4)	0.0258 (3)	
H12	0.0095	0.7978	0.0168	0.031*	
C13	−0.1635 (2)	0.95325 (18)	0.06335 (4)	0.0267 (3)	
H13A	−0.3154	0.9416	0.0767	0.032*	
H13B	−0.1645	1.0637	0.0474	0.032*	
C14	0.0378 (2)	0.98096 (17)	0.09963 (4)	0.0259 (3)	
H14A	0.0112	1.0894	0.1208	0.031*	
H14B	0.1881	1.0071	0.0867	0.031*	
C15	−0.3433 (3)	0.7554 (2)	−0.00528 (4)	0.0344 (3)	
H15A	−0.4931	0.7358	0.0077	0.052*	
H15B	−0.3195	0.6475	−0.0263	0.052*	
H15C	−0.3470	0.8664	−0.0205	0.052*	
C16	0.5868 (3)	0.3403 (2)	0.13812 (4)	0.0324 (3)	
H16A	0.4446	0.2788	0.1474	0.049*	
H16B	0.6262	0.2756	0.1101	0.049*	
H16C	0.5582	0.4693	0.1345	0.049*	
C17	0.7511 (2)	0.41843 (16)	0.21210 (4)	0.0213 (3)	
C18	0.5547 (2)	0.51267 (16)	0.22355 (4)	0.0215 (3)	
H18	0.4286	0.5234	0.2021	0.026*	
C19	0.5460 (2)	0.59047 (16)	0.26667 (4)	0.0214 (3)	
H19	0.4111	0.6529	0.2747	0.026*	
C20	0.7298 (2)	0.57948 (16)	0.29851 (4)	0.0195 (3)	
C21	0.9266 (2)	0.48535 (16)	0.28615 (4)	0.0214 (3)	
H21	1.0549	0.4774	0.3073	0.026*	
C22	0.9355 (2)	0.40431 (16)	0.24356 (4)	0.0219 (3)	
H22	1.0681	0.3386	0.2357	0.026*	
C23	0.7117 (2)	0.67370 (17)	0.34374 (4)	0.0223 (3)	
C24	0.9260 (2)	0.69855 (17)	0.37672 (4)	0.0216 (3)	
H24	1.0706	0.7109	0.3605	0.026*	
C25	0.9183 (2)	0.87238 (17)	0.40970 (4)	0.0242 (3)	
H25A	0.9196	0.9833	0.3939	0.029*	
H25B	0.7700	0.8658	0.4244	0.029*	
C26	1.1281 (2)	0.89120 (17)	0.44422 (4)	0.0251 (3)	
H26A	1.1156	1.0027	0.4654	0.030*	
H26B	1.2757	0.9082	0.4297	0.030*	
C27	1.1396 (2)	0.72105 (18)	0.46893 (4)	0.0252 (3)	
H27	0.9918	0.7093	0.4842	0.030*	
C28	1.1465 (2)	0.54731 (17)	0.43608 (4)	0.0250 (3)	
H28A	1.2954	0.5536	0.4216	0.030*	
H28B	1.1451	0.4369	0.4521	0.030*	
C29	0.9384 (2)	0.52528 (17)	0.40108 (4)	0.0244 (3)	
H29A	0.7900	0.5053	0.4151	0.029*	
H29B	0.9552	0.4151	0.3797	0.029*	
C30	1.3480 (2)	0.74087 (19)	0.50368 (4)	0.0329 (3)	
H30A	1.3497	0.6296	0.5188	0.049*	
H30B	1.3330	0.8497	0.5250	0.049*	
H30C	1.4951	0.7561	0.4896	0.049*	

### Atomic displacement parameters (Å^2^)

**Table:**

	*U*^11^	*U*^22^	*U*^33^	*U*^12^	*U*^13^	*U*^23^
O1	0.0278 (5)	0.0285 (5)	0.0217 (5)	0.0011 (4)	0.0032 (4)	−0.0006 (4)
O2	0.0202 (5)	0.0442 (6)	0.0272 (5)	0.0045 (4)	0.0047 (4)	0.0015 (4)
O3	0.0290 (5)	0.0311 (5)	0.0217 (5)	0.0059 (4)	0.0004 (4)	−0.0002 (4)
O4	0.0217 (5)	0.0480 (6)	0.0301 (6)	0.0073 (5)	0.0046 (4)	−0.0009 (5)
C1	0.0333 (8)	0.0294 (7)	0.0232 (7)	0.0005 (6)	−0.0047 (6)	−0.0016 (6)
C2	0.0240 (7)	0.0176 (6)	0.0220 (7)	0.0046 (5)	0.0028 (6)	0.0040 (5)
C3	0.0187 (7)	0.0193 (6)	0.0270 (7)	−0.0002 (5)	−0.0017 (5)	0.0042 (5)
C4	0.0172 (7)	0.0214 (6)	0.0259 (7)	0.0016 (5)	0.0025 (5)	0.0058 (5)
C5	0.0175 (7)	0.0183 (6)	0.0227 (7)	0.0032 (5)	0.0009 (5)	0.0058 (5)
C6	0.0173 (7)	0.0178 (6)	0.0263 (7)	0.0000 (5)	0.0012 (5)	0.0035 (5)
C7	0.0197 (7)	0.0195 (6)	0.0256 (7)	0.0000 (5)	0.0050 (5)	0.0055 (5)
C8	0.0217 (7)	0.0223 (6)	0.0224 (7)	0.0027 (5)	0.0018 (6)	0.0053 (5)
C9	0.0182 (7)	0.0279 (7)	0.0197 (7)	0.0013 (5)	0.0024 (5)	0.0022 (5)
C10	0.0285 (8)	0.0238 (6)	0.0288 (8)	0.0031 (6)	−0.0009 (6)	0.0025 (6)
C11	0.0295 (8)	0.0265 (7)	0.0269 (8)	0.0009 (6)	−0.0016 (6)	−0.0018 (6)
C12	0.0243 (7)	0.0334 (7)	0.0189 (7)	0.0012 (6)	0.0026 (6)	0.0001 (6)
C13	0.0281 (8)	0.0282 (7)	0.0241 (7)	0.0049 (6)	0.0000 (6)	0.0043 (6)
C14	0.0267 (7)	0.0245 (7)	0.0253 (7)	0.0021 (6)	−0.0017 (6)	0.0000 (6)
C15	0.0362 (9)	0.0414 (8)	0.0234 (8)	0.0020 (7)	−0.0038 (6)	−0.0001 (6)
C16	0.0377 (9)	0.0345 (8)	0.0233 (8)	0.0044 (7)	−0.0038 (6)	−0.0001 (6)
C17	0.0237 (7)	0.0187 (6)	0.0214 (7)	−0.0014 (5)	0.0034 (6)	0.0032 (5)
C18	0.0194 (7)	0.0209 (6)	0.0240 (7)	−0.0002 (5)	−0.0025 (5)	0.0057 (5)
C19	0.0177 (7)	0.0194 (6)	0.0279 (7)	0.0008 (5)	0.0035 (6)	0.0050 (5)
C20	0.0174 (6)	0.0188 (6)	0.0224 (7)	−0.0012 (5)	0.0022 (5)	0.0047 (5)
C21	0.0184 (7)	0.0227 (6)	0.0236 (7)	0.0008 (5)	0.0004 (5)	0.0064 (5)
C22	0.0201 (7)	0.0195 (6)	0.0273 (7)	0.0030 (5)	0.0050 (6)	0.0052 (5)
C23	0.0205 (7)	0.0226 (6)	0.0247 (7)	0.0009 (5)	0.0046 (6)	0.0055 (5)
C24	0.0196 (7)	0.0244 (6)	0.0209 (7)	0.0009 (5)	0.0041 (5)	0.0022 (5)
C25	0.0244 (7)	0.0235 (6)	0.0247 (7)	0.0026 (5)	0.0034 (6)	0.0019 (5)
C26	0.0255 (7)	0.0250 (6)	0.0237 (7)	0.0001 (5)	0.0023 (6)	−0.0015 (5)
C27	0.0245 (7)	0.0298 (7)	0.0208 (7)	0.0002 (6)	0.0033 (6)	0.0012 (6)
C28	0.0275 (8)	0.0251 (6)	0.0224 (7)	0.0028 (6)	0.0005 (6)	0.0033 (5)
C29	0.0260 (7)	0.0239 (6)	0.0226 (7)	−0.0002 (5)	0.0013 (6)	0.0012 (5)
C30	0.0342 (9)	0.0367 (8)	0.0262 (8)	0.0037 (7)	−0.0020 (6)	−0.0006 (6)

### Geometric parameters (Å, º)

**Table:**

O1—C2	1.3658 (15)	C15—H15A	0.9800
O1—C1	1.4289 (16)	C15—H15B	0.9800
O2—C8	1.2273 (14)	C15—H15C	0.9800
O3—C17	1.3663 (15)	C16—H16A	0.9800
O3—C16	1.4290 (16)	C16—H16B	0.9800
O4—C23	1.2269 (14)	C16—H16C	0.9800
C1—H1A	0.9800	C17—C22	1.3898 (18)
C1—H1B	0.9800	C17—C18	1.3936 (17)
C1—H1C	0.9800	C18—C19	1.3860 (17)
C2—C7	1.3897 (18)	C18—H18	0.9500
C2—C3	1.3935 (17)	C19—C20	1.3908 (17)
C3—C4	1.3863 (17)	C19—H19	0.9500
C3—H3	0.9500	C20—C21	1.4022 (17)
C4—C5	1.3910 (17)	C20—C23	1.4915 (17)
C4—H4	0.9500	C21—C22	1.3785 (17)
C5—C6	1.4000 (16)	C21—H21	0.9500
C5—C8	1.4886 (17)	C22—H22	0.9500
C6—C7	1.3806 (17)	C23—C24	1.5152 (18)
C6—H6	0.9500	C24—C25	1.5317 (16)
C7—H7	0.9500	C24—C29	1.5427 (17)
C8—C9	1.5142 (18)	C24—H24	1.0000
C9—C10	1.5251 (17)	C25—C26	1.5264 (18)
C9—C14	1.5418 (17)	C25—H25A	0.9900
C9—H9	1.0000	C25—H25B	0.9900
C10—C11	1.5290 (18)	C26—C27	1.5289 (17)
C10—H10A	0.9900	C26—H26A	0.9900
C10—H10B	0.9900	C26—H26B	0.9900
C11—C12	1.5266 (18)	C27—C30	1.5244 (18)
C11—H11A	0.9900	C27—C28	1.5283 (17)
C11—H11B	0.9900	C27—H27	1.0000
C12—C15	1.5252 (18)	C28—C29	1.5277 (17)
C12—C13	1.5251 (18)	C28—H28A	0.9900
C12—H12	1.0000	C28—H28B	0.9900
C13—C14	1.5256 (18)	C29—H29A	0.9900
C13—H13A	0.9900	C29—H29B	0.9900
C13—H13B	0.9900	C30—H30A	0.9800
C14—H14A	0.9900	C30—H30B	0.9800
C14—H14B	0.9900	C30—H30C	0.9800
			
C2—O1—C1	117.19 (10)	H15B—C15—H15C	109.5
C17—O3—C16	117.13 (10)	O3—C16—H16A	109.5
O1—C1—H1A	109.5	O3—C16—H16B	109.5
O1—C1—H1B	109.5	H16A—C16—H16B	109.5
H1A—C1—H1B	109.5	O3—C16—H16C	109.5
O1—C1—H1C	109.5	H16A—C16—H16C	109.5
H1A—C1—H1C	109.5	H16B—C16—H16C	109.5
H1B—C1—H1C	109.5	O3—C17—C22	115.81 (11)
O1—C2—C7	115.38 (11)	O3—C17—C18	124.01 (12)
O1—C2—C3	124.43 (12)	C22—C17—C18	120.19 (12)
C7—C2—C3	120.18 (12)	C19—C18—C17	118.95 (12)
C4—C3—C2	118.62 (12)	C19—C18—H18	120.5
C4—C3—H3	120.7	C17—C18—H18	120.5
C2—C3—H3	120.7	C18—C19—C20	121.69 (11)
C3—C4—C5	122.11 (11)	C18—C19—H19	119.2
C3—C4—H4	118.9	C20—C19—H19	119.2
C5—C4—H4	118.9	C19—C20—C21	118.35 (12)
C4—C5—C6	118.18 (12)	C19—C20—C23	118.46 (11)
C4—C5—C8	118.67 (11)	C21—C20—C23	123.16 (12)
C6—C5—C8	123.13 (12)	C22—C21—C20	120.54 (12)
C7—C6—C5	120.43 (12)	C22—C21—H21	119.7
C7—C6—H6	119.8	C20—C21—H21	119.7
C5—C6—H6	119.8	C21—C22—C17	120.26 (11)
C6—C7—C2	120.42 (11)	C21—C22—H22	119.9
C6—C7—H7	119.8	C17—C22—H22	119.9
C2—C7—H7	119.8	O4—C23—C20	119.73 (12)
O2—C8—C5	119.42 (12)	O4—C23—C24	120.28 (12)
O2—C8—C9	119.92 (11)	C20—C23—C24	119.99 (10)
C5—C8—C9	120.59 (10)	C23—C24—C25	111.55 (10)
C8—C9—C10	112.24 (10)	C23—C24—C29	109.11 (11)
C8—C9—C14	106.83 (11)	C25—C24—C29	110.09 (10)
C10—C9—C14	110.54 (11)	C23—C24—H24	108.7
C8—C9—H9	109.1	C25—C24—H24	108.7
C10—C9—H9	109.1	C29—C24—H24	108.7
C14—C9—H9	109.1	C26—C25—C24	111.27 (10)
C9—C10—C11	111.49 (10)	C26—C25—H25A	109.4
C9—C10—H10A	109.3	C24—C25—H25A	109.4
C11—C10—H10A	109.3	C26—C25—H25B	109.4
C9—C10—H10B	109.3	C24—C25—H25B	109.4
C11—C10—H10B	109.3	H25A—C25—H25B	108.0
H10A—C10—H10B	108.0	C25—C26—C27	111.83 (11)
C12—C11—C10	111.75 (11)	C25—C26—H26A	109.2
C12—C11—H11A	109.3	C27—C26—H26A	109.3
C10—C11—H11A	109.3	C25—C26—H26B	109.3
C12—C11—H11B	109.3	C27—C26—H26B	109.3
C10—C11—H11B	109.3	H26A—C26—H26B	107.9
H11A—C11—H11B	107.9	C30—C27—C28	111.83 (10)
C15—C12—C13	111.66 (11)	C30—C27—C26	111.76 (11)
C15—C12—C11	111.45 (12)	C28—C27—C26	109.44 (11)
C13—C12—C11	109.48 (11)	C30—C27—H27	107.9
C15—C12—H12	108.0	C28—C27—H27	107.9
C13—C12—H12	108.0	C26—C27—H27	107.9
C11—C12—H12	108.0	C29—C28—C27	112.45 (10)
C12—C13—C14	112.18 (10)	C29—C28—H28A	109.1
C12—C13—H13A	109.2	C27—C28—H28A	109.1
C14—C13—H13A	109.2	C29—C28—H28B	109.1
C12—C13—H13B	109.2	C27—C28—H28B	109.1
C14—C13—H13B	109.2	H28A—C28—H28B	107.8
H13A—C13—H13B	107.9	C28—C29—C24	111.02 (11)
C13—C14—C9	111.44 (11)	C28—C29—H29A	109.4
C13—C14—H14A	109.3	C24—C29—H29A	109.4
C9—C14—H14A	109.3	C28—C29—H29B	109.4
C13—C14—H14B	109.3	C24—C29—H29B	109.4
C9—C14—H14B	109.3	H29A—C29—H29B	108.0
H14A—C14—H14B	108.0	C27—C30—H30A	109.5
C12—C15—H15A	109.5	C27—C30—H30B	109.5
C12—C15—H15B	109.5	H30A—C30—H30B	109.5
H15A—C15—H15B	109.5	C27—C30—H30C	109.5
C12—C15—H15C	109.5	H30A—C30—H30C	109.5
H15A—C15—H15C	109.5	H30B—C30—H30C	109.5
			
C1—O1—C2—C7	173.31 (10)	C16—O3—C17—C22	177.02 (11)
C1—O1—C2—C3	−7.90 (17)	C16—O3—C17—C18	−3.19 (17)
O1—C2—C3—C4	179.14 (11)	O3—C17—C18—C19	179.81 (11)
C7—C2—C3—C4	−2.13 (17)	C22—C17—C18—C19	−0.41 (18)
C2—C3—C4—C5	1.44 (17)	C17—C18—C19—C20	1.11 (18)
C3—C4—C5—C6	0.71 (17)	C18—C19—C20—C21	−0.56 (18)
C3—C4—C5—C8	−177.36 (10)	C18—C19—C20—C23	177.42 (11)
C4—C5—C6—C7	−2.21 (17)	C19—C20—C21—C22	−0.68 (18)
C8—C5—C6—C7	175.77 (11)	C23—C20—C21—C22	−178.56 (11)
C5—C6—C7—C2	1.54 (17)	C20—C21—C22—C17	1.37 (18)
O1—C2—C7—C6	179.51 (10)	O3—C17—C22—C21	178.99 (11)
C3—C2—C7—C6	0.67 (17)	C18—C17—C22—C21	−0.81 (18)
C4—C5—C8—O2	21.98 (17)	C19—C20—C23—O4	12.43 (18)
C6—C5—C8—O2	−155.99 (12)	C21—C20—C23—O4	−169.69 (12)
C4—C5—C8—C9	−155.03 (11)	C19—C20—C23—C24	−167.66 (11)
C6—C5—C8—C9	27.00 (17)	C21—C20—C23—C24	10.22 (18)
O2—C8—C9—C10	39.30 (16)	O4—C23—C24—C25	−29.70 (16)
C5—C8—C9—C10	−143.71 (11)	C20—C23—C24—C25	150.39 (11)
O2—C8—C9—C14	−82.01 (14)	O4—C23—C24—C29	92.15 (14)
C5—C8—C9—C14	94.98 (13)	C20—C23—C24—C29	−87.76 (13)
C8—C9—C10—C11	−173.66 (11)	C23—C24—C25—C26	177.09 (10)
C14—C9—C10—C11	−54.51 (14)	C29—C24—C25—C26	55.81 (14)
C9—C10—C11—C12	56.90 (15)	C24—C25—C26—C27	−57.41 (14)
C10—C11—C12—C15	179.37 (10)	C25—C26—C27—C30	−179.46 (10)
C10—C11—C12—C13	−56.60 (14)	C25—C26—C27—C28	56.11 (14)
C15—C12—C13—C14	−179.84 (11)	C30—C27—C28—C29	179.88 (11)
C11—C12—C13—C14	56.25 (14)	C26—C27—C28—C29	−55.74 (14)
C12—C13—C14—C9	−55.65 (14)	C27—C28—C29—C24	56.02 (14)
C8—C9—C14—C13	176.27 (10)	C23—C24—C29—C28	−177.67 (10)
C10—C9—C14—C13	53.89 (13)	C25—C24—C29—C28	−54.94 (13)

### Hydrogen-bond geometry (Å, º)

**Table:**

*D*—H···*A*	*D*—H	H···*A*	*D*···*A*	*D*—H···*A*
C9—H9···O2^i^	1.00	2.57	3.4694 (18)	150
C24—H24···O4^ii^	1.00	2.60	3.5244 (18)	154

Symmetry codes: (i) *x*−1, *y*, *z*; (ii) *x*+1, *y*, *z*.
